# A National Survey of Skin Infections, Care Behaviors and MRSA Knowledge in the United States

**DOI:** 10.1371/journal.pone.0104277

**Published:** 2014-08-19

**Authors:** Jocelyn R. Wilder, Duane T. Wegener, Michael Z. David, Charles Macal, Robert Daum, Diane S. Lauderdale

**Affiliations:** 1 The Department of Health Studies, The University of Chicago, Chicago, Illinois, United States of America; 2 The Department of Psychology, The Ohio State University, Columbus, Ohio, United States of America; 3 Decision and Information Sciences Division, Argonne National Laboratory, Argonne, Illinois, United States of America; Arizona State University, United States of America

## Abstract

A nationally representative sample of approximately 2000 individuals was surveyed to assess SSTI infections over their lifetime and then prospectively over six-months. Knowledge of MRSA, future likelihood to self-treat a SSTI and self-care behaviors was also queried. Chi square tests, linear and multinomial regression were used for analysis. About 50% of those with a reported history of a SSTI typical of MRSA had sought medical treatment. MRSA knowledge was low: 28% of respondents could describe MRSA. Use of protective self-care behaviors that may reduce transmission, such as covering a lesion, differed with knowledge of MRSA and socio-demographics. Those reporting a history of a MRSA-like SSTI were more likely to respond that they would self-treat than those without such a history (OR 2.05 95% CI 1.40, 3.01; p<0.001). Since half of respondents reported not seeking care for past lesions, incidence determined from clinical encounters would greatly underestimate true incidence. MRSA knowledge was not associated with seeking medical care, but was associated with self-care practices that may decrease transmission.

## Introduction

The number of outpatient visits for medical care in the United States for a skin or soft tissue infection (SSTI) is estimated to have risen from 8.6 million in 1997 to 14.2 million in 2005, a 65% increase [1], [2]. The increase is likely related to the appearance of new strains of methicillin-resistant *Staphylococcus aureus* (MRSA) spreading among healthy people in the United States. These new strains, recognized in the late 1990s, as Community-Associated MRSA (CA-MRSA), are distinct from older strains of MRSA which were usually confined to healthcare settings and typically infecting patients with previous health care exposures. While SSTIs may be caused by various conditions the majority of CA-MRSA infections are SSTIs that present as either abscesses or cellulitis. CA-MRSA strains likely account for a majority of SSTIs among ambulatory patients in recent years [3]–[7].

Data on the incidence of SSTIs in the United States are not systematically collected. Even if collected, such data would not represent the true incidence of SSTIs in the general population because we think it is likely that not all SSTIs are brought to medical attention. The changing incidence in SSTIs observed in clinical settings might reflect trends in the overall incidence in the population, but it is also possible that a change in the incidence of clinically attended SSTIs reflects a change in the proportion of individuals with SSTIs who seek medical care. Media attention to CA-MRSA has likely raised popular awareness of the risks of progression of MRSA SSTIs to invasive infections. For example, there was significant media attention and public interest focused on CA-MRSA after a high profile medical publication estimated that in the United States more individuals had died from MRSA than AIDS in 2005 [8]–[10]. Therefore, it is not certain from current data how prevalent MRSA infections (or SSTIs in general) are in the population, what proportion of such infections present in clinical settings, or whether trends in clinically attended skin infections may be due in part to heightened awareness of MRSA.

We designed a survey to assess how frequently adults in a nationally representative sample reported having ever had a lesion similar to one described in a vignette that was typical of a CA-MRSA SSTI. After six months, respondents were resurveyed and asked about occurrence of described lesion in the six months between the survey waves. For those who reported having a lesion, we asked whether they had sought medical care. The purpose of this study was threefold: (1) to estimate the lifetime prevalence and six-month occurrence of an SSTI similar to a CA-MRSA skin infection; (2) to estimate the proportion of such skin infections brought to medical attention; and (3) to assess whether a past sore and/or knowledge of MRSA influences care-seeking and treatment behaviors, both actual and intended.

## Methods

The survey was supported by the Time-Sharing Experiments for the Social Sciences (TESS) program with funding from the National Science Foundation. Analysis was support by the National Institute of General Medical Sciences (NIGMS). The survey (File S1) was conducted by Knowledge Network and distributed to their KnowledgePanel. KnowledgePanel is a probability-based Internet panel with a sampling frame of approximately 50,000 individuals (ages 18 and older) who are sampled from the United States Postal Service's Computerized Delivery Sequence file, which comprises approximately 97percent of the physical addresses in all 50 states. Further details for Knowledge Network's Panel design are available at www.knowledgenetworks.com/knpanel/docs/knowledgepanel(R)-design-summary-description.pdf Basic demographic data are collected from all participants when they join the panel. In this study, African American and Hispanic households were oversampled in order to ensure a sufficient sample size to assess differences across major racial/ethnic groups. The data received from Knowledge Network was de-identified. The University of Chicago and The University of Ohio IRBs approved the research.

The initial survey was completed by 1980 adults in August 2011. Six months later, 1379 of the participants in the initial sample completed the follow-up survey.

**Figure 1:**
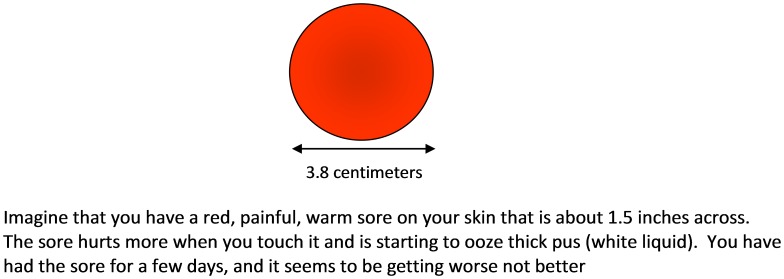
Description of Draining Sore Vignette.

**Figure 2:**
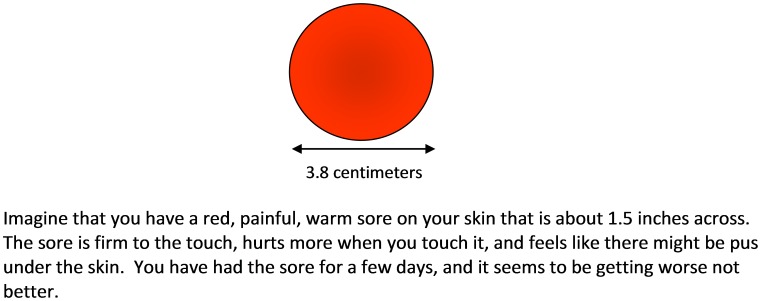
Description of Non-Draining Sore Vignette.

The descriptions of the lesions represented in the vignettes are typical of abscesses caused by MRSA. The description represents a moderately severe infection and was used to elicit care seeking for a level of severity that a physician would recommend clinical care. At baseline, participants were randomly assigned to receive one of two vignettes that varied in one respect. In one version the SSTI was described as draining pus (Figure 1); it was thought that presence of a draining lesion may influence one's care seeking and self-care behaviors, and in the other vignette, pus was mentioned but the sore was not described as draining (Figure 2). Note that a draining SSTI would have passed through a non-draining phase first, whereas not all non-draining SSTIs progress to a draining phase.

After the description, respondents were asked if they had ever had an SSTI similar to that described and whether they had sought medical care. All respondents were then asked how likely they would be to self-treat such an infection if they experienced it now, using a 9 point rating scale ranging from 1 (not at all likely to self-treat) to 9 (very likely to self-treat). For analysis, self-treatment was collapsed into three levels: 1 (not at all likely to self-treat) through 3 were categorized as likely to seek care, 4 through 6 were categorized as equally likely to seek care or self-treat and 7 through 9 (very likely to self-treat) were categorized as likely to self-treat.

Respondents were asked whether they would use each of three self-care behaviors that may alter the likelihood of MRSA transmission to others: “covering the sore” and “avoid sharing personal items like towels with household members,” both of which should decrease risk of transmission, and “try to squeeze out the pus” which may spread bacteria and increase risk of transmission. Respondents were then asked whether they had heard of MRSA and whether they knew what MRSA was. If a respondent indicated that they knew what MRSA was, a free-text box was provided for them to define MRSA. We used the responses to these questions to assign individuals to three mutually exclusive groups: those who had not heard of MRSA (“unaware”), those who had heard of it but did not know what it was (“aware”) and those who could define MRSA (“knowledge”). Those in the aware and knowledge groups were asked about the likelihood of the SSTI described earlier being caused by MRSA, using a 9 point rating scale from 1 (not at all likely to be MRSA) to 9 (very likely to be MRSA). They were also asked how many people they knew who had had a MRSA infection.

There was an experiment also embedded in the survey. A random half of the respondents received an informational message based on CDC's MRSA website information. The following questions were asked of those receiving and not receiving the informational message: (1) How likely would you be to seek medical care if you had a sore similar to the one described, with responses ranging from 1 (not at all likely) to 9 (very likely). (2) If left untreated, the consequences of the described sore are: 1 (not serious) to 9 (very serious). (3) How likely is the described sore to be MRSA: 1 (not at all likely) to 9 (very likely). Six months after the initial survey all respondents (both those who had received the MRSA information and those who had not) received a follow-up survey with the same sore description. They were asked if during the prior six months they had had a sore similar to the one described and whether they had sought medical care.

## Data Analysis

The proportions of those with a lifetime and six-month history of SSTIs similar to the description were determined for all respondents and stratified by demographics and other characteristics. Variation in seeking medical care was examined by individual characteristics, including knowledge of MRSA. Variation in intention to self-treat and use of self-care behaviors were examined by lesion history, socio-demographics, and MRSA knowledge. Significance in differences between groups was assessed by chi-square tests for categorical and dichotomous variables and by linear regression for continuous variables. Age and income were tested for linear trend in proportions using linear regression. Covariates that were significantly associated with the dependent or exposure variables at *p*<0.10 were included in the multivariable models.

Logistic regression was used to identify factors related to having had a previous sore. A multinomial logistic regression model was used to determine whether history of a previous lesion was associated independently either with intention to seek medical care or with intention to self-treat, each compared to those who responded in the middle of the scale (noncommittal as to whether they would self-treat or not). The model is adjusted for characteristics associated with care-seeking. All analyses used sampling weights to adjust raw responses and generate estimates representative of the U.S. population. Data were analyzed in STATA 12, and all analyses use variances for weighted survey data.

## Results

Demographic characteristics of the initial sample of 1980 adult respondents and the 1376 that completed the six-month follow-up survey are presented in Table 1. Those that participated in the six-month follow-up had similar demographic characteristics to those who participated in the initial survey. Although the informational message that half of respondents received at the end of the initial survey had an immediate influence on perceptions of the severity of a SSTI, compared to those who did not receive the message, (mean 7.67 vs. 7.08, F = 18.09, *p*<0.001) and on the likelihood that a sore such as that described was MRSA (mean 6.36 vs. 5.54, F = 58.24, *p*<0.001), neither effects persisted over the 6-month period. Nor was there any difference in care seeking for those who had a sore in the six months between the surveys (χ2 = 0.03, *p* = 0.87). Therefore, we have combined all responses from the six-month follow up in this report.

**Table 1:** Demographic Characteristics of Survey Respondents, Weighted to Reflect the National Population.

	Initial survey		Follow-up survey	
Variables	Weighted % (N)	95% CI	Weighted % (N)	95% CI
**All**	100.0 (1980)		100.0 (1376)	
***SOCIODEMOGRAPHIC FACTORS***				
**Gender**				
Male	48.3 (964)	45.5, 51.2	49.8 (689)	46.4, 53.2
Female	51.7 (1016)	48.8, 54.5	50.2 (687)	46.8, 53.6
**Age, years**				
18–29	21.7 (355)	19.9, 24.3	19.8 (216)	17.0, 23.0
30–44	26.0 (508)	23.6, 28.6	24.5 (333)	21.7, 27.5
45–59	27.7 (602)	25.3, 30.2	28.5 (436)	25.7 31.6
≥60	24.6 (515)	22.3, 27.1	27.2 (391)	24.3, 30.2
**Race/Ethnicity**				
Non-Hispanic White	67.9 (1077)	65.4, 70.3	69.7 (789)	66.7, 72.5
Non-Hispanic Black	11.5 (406)	10,2, 12.9	10.4 (262)	8.9, 12.0
Hispanic	13.9 (409)	12.3, 15.7	13.4 (265)	11.5, 15.5
Non-Hispanic Other	6.7 (88)	5.2, 8.5	6.5 (60)	4.9, 8.8
**Education**				
≤High school	43.7 (890)	40.9, 46.5	42.4 (612)	54.3, 60.9
>High school	56.3 (1090)	53.3, 59.1	57.6 (764)	39.1, 45.7
**Household Income**				
<20 k	16.3 (329)	14.4, 18.5	14.1 (199)	11.9. 16.5
20 k-<40 k	23.1 (429)	20..7, 25.6	23.8 (289)	21.0, 26.9
40 k-<60 k	16.8 (358)	14.7, 19.1	16.3 (232)	14,0, 19.0
≥60 k	894 (43.8)	41.0, 46.6	45.8 (656)	42.2, 49.2
**Geography**				
Non metropolitan area	16.1 (286)	14.1, 18.3	15.9 (197)	13.5, 18.5
Metropolitan area	83.9 (1694)	81.7, 85.9	84.1 (1179)	81.5, 86.5
**Children in Home**				
No children	67.3(1,286)	64.6, 69.8	69.7 (935)	66.6, 72.7
Children	32.7 (694)	30.2, 35.4	30.3 (441)	27.3, 33.4
**Health care provider**				
No regular provider	20.4 (366)	18.1, 22.9	21.2 (253)	18.4, 24.3
Regular provider	79.6 (1589)	77.1, 81.9	78.8 (1109)	75.7, 81.6
***MRSA-RELATED FACTORS FROM INITIAL SURVEY***				
**Knowledge of MRSA**				
MRSA unaware	46.7 (921)	43.9, 49.0	46.7 (643)	43.3, 50.1
MRSA aware	24.9 (486)	22.5, 27.4	25.7 (346)	22.8, 28.7
MRSA knowledge	28.3 (28.3)	25.9, 30.9	27.6 (393)	24.7, 30.7
**Knew someone who had MRSA**				
Does not know	59.9 (633)	56.0, 63.6	57.6 (436)	53.0, 62.1
Knows someone	40.1 (415)	36.4, 44.0	42.4 (302)	37.9, 47.0

TESS Survey 2011.

### Lifetime Prevalence and Incidence of SSTIs Typical of CA-MRSA

Table 2 displays the lifetime prevalence and six month incidence of lesions. Thirteen percent of respondents reported having had a lesion similar to the one described prior to the survey, 53% of those with a previous lesion responded that they had not sought medical care. Respondents with lower household income were more likely to respond that they had a previous lesion (*p^trend^*<0.001) than those with higher household incomes. In the follow-up survey, 6% of respondents reported having had an SSTI in the six months between the initial and the follow-up survey. Among those with an SSTI in this interval, 48 percent had not sought medical treatment. In the logistic regression displayed in Table 3, after adjusting for, gender, age, race/ethnicity, education, income and having a regular health care provider, significant factors associated with reporting a previous sore were having a household income below 60,000 (*p*<0.05) and having a regular care provider (*p* = 0.03). Hispanic race was marginally significant (*p* = 0.05). In the second wave, Hispanics were more likely to respond that they had had a lesion in the past six months than Whites (*p*<0.001).

**Table 2:** Lifetime Prevalence and Subsequent Six-month Occurrence of SSTIs similar to CA-MRSA by Selected Characteristics.

	Lifetime Prevalence (%)	95% CI	*p* value	6 month Prevalence (%)	95% CI	*p*-value
**All**	13.5	11.7, 15.5		6.2	4.8, 8.0	
Sought Medical Care	47.2	39.8, 54.7		52.0	38.4, 64.7	
Self-Care	53.0	45.3, 60.3		48.0	35.3, 61.6	
***SOCIO-DEMOGRAPHIC FACTORS***						
**Gender**			*0.*95			0.65
Male	13.5	11.2, 16.5		5.8	4.0, 8.3	
Female	13.4	11.0, 16.3		6.5	4.5, 9.3	
**Race/Ethnicity**			*0.*07			<0.001
Non-Hispanic White	11.8	9.7, 14.2		4.1	2.7, 6.2	
Non-Hispanic Black	16.3	12.3, 21.3		6.7	4.1, 10.7	
Hispanic	18.3	13.8, 23.7		11.8	7.7, 17.6	
Non-Hispanic Other	16.3	8.8, 18.2		14.8	6.5, 29.0	
**Age, years**			*0.*67			0.22
18–29	14.0	10.0, 19.3		8.1	4.6, 13.9	
30–44	14.9	11.4, 19.2		7.7	4.9, 12.0	
45–59	13.4	10.4, 17.1		4.5	2.9, 6.9	
≥60	11.6	8.6, 15.5		4.7	2.7, 8.2	
**Education**			*0.*73			0.99
≤High school	13.9	11.2, 17.0		6.2	4.1, 9.3	
>High school	13.2	10.9, 15.9		6.2	4.5, 8.5	
**Income**			*p^trend^*<0.01			*p^trend^* = 0.06
<20 k	17.3	12.9, 22.8		10.7	6.5, 17.3	
20 K-<40 k	14.8	11.0, 19.7		7.0	4.2, 11.3	
40 k-<60 k	16.9	12.3, 22.8		4.0	1.8, 8.9	
≥60 k	10.0	7.9, 12.8		5.0	3.2, 7.5	
**Geography**			0.90			0.41
Non metropolitan area	2.2	1.5, 3.3		1.7		
Metropolitan area	11.5	9.6, 13.1		11.2		

TESS Survey 2011.

**Table 3:** Logistic Regression Analysis for Factors associated with having a previous sore.

Variables				
	Unadjusted	*p*-values	Adjusted	*p*-value
	OR (95% CI)		OR (95% CI)	
**Gender**				
Male	1.00		1.00	
Female	0.99 (0.12, 0.20)	0.96	0.94 (0.98, 1.31)	0.73
**Age (per year)**	1.00 (0.99, 1.01)	0.38	0.99 (0.98, 1.01	0.26
**Race**				
White	1.00 (Ref)		1.00 (Ref)	
Black	1.47 (0.99, 2.18)	0.05	1.30 (0.83, 1.97)	0.26
Hispanic	1.67 (1.13, 2.49)	0.01	1.53 (0.99, 2.36)	0.05
Other	1.46 (0.70, 3.05)	0.31	1.46 (0.69, 3.07)	0.33
**Education**				
<High School	1.00 (Ref)		1.00 (Ref)	
High School graduate plus	0.94 (0.68, 1.31)	0.83	1.17 (0.80, 1.71)	0.41
**Income**				
<20 k	1.87 (1.21, 2.91)	0.01	1.96 (1.18, 3.26)	0.01
20 k-<40 k	1.56 (1.00, 2.41)	0.05	1.66 (1.03, 2.62	0.04
40 k-<60 k	1.83 (1.15, 2.89)	0.01	1.92 (1.20, 3.06)	0.01
≥60 k	1.00 (Ref)		1.00 (Ref)	
**Health Care Provider**				
No Regular Health Care Provider	1.00 (Ref)		1.00 (Ref)	
Regular Health Care Provider	1.38 (0.88, 2.18)	0.16	1.72 (1.05, 2.81)	0.03

### Associations with MRSA Knowledge

 MRSA knowledge varied significantly by gender, race/ethnicity, age, household income and education (Table 4). The majority of respondents reported that they had heard of MRSA (53%); approximately half of those (28% of all respondents) could define MRSA. Since definitions were almost all plausible, we did not divide them into more or less correct ones. Correlates of awareness and knowledge were similar. Women, Whites, and those with higher education and greater income were all more likely to have both awareness and knowledge.

**Table 4:** Demographic Characteristics by Knowledge of MRSA.

Variables	MRSA UNAWARE (N = 921)		MRSA AWARENESS^a^ (N = 486)		MRSA KNOWLEDGE^b^ (N = 565)		
	Weighted distribution (%)	95% CI	Weighted distribution (%)	95% CI	Weighted distribution (%)	95% CI	*p*-value
**All**	46.7	43.9, 49.6	24.7	22.5, 27.4	28.6	25.9, 30.9	
***SOCIO-DEMOGRAPHIC FACTORS***							
**Gender**							<0.003
Male	50.1	46.1, 54.2	26.1	22.7, 29.9	23.8	20.5, 27.4	
Female	43.6	39.8, 47.6	23.8	20.5, 27.3	32.6	29.1, 36.4	
**Race/Ethnicity**							<0.001
Non-Hispanic White	40.5	37.0, 44.1	26.6	23.5, 29.9	32.9	29.7, 36.4	
Non-Hispanic Black	51.5	45.3, 57.5	26.6	21.5, 32.5	21.9	17.4, 27.2	
Hispanic	67.6	61.6, 73.1	18.1	14.0, 23.0	14.3	10.4, 19.4	
Non-Hispanic Other	58.7	46.1, 70.2	19.4	11.4, 31.1	21.9	13.6, 33.3	
**Age, years**							0.007
18–29	48.0	40.4, 54.8	28.1	22.3, 34.6	23.9	18.4, 30.4	
30–44	54.0	48.5, 59.3	19.1	15.2, 23.7	27.0	22.6, 31.9	
45–59	41.6	36.6, 46.7	24.9	20.7, 29.5	33.6	28.9, 38.5	
≥60	43.8	38.4, 49.4	28.3	23.7, 33.5	27.9	23.3, 33.0	
**Education**							<.001
≤High school	52.5	48.3, 56.7	26.8	23.2, 30.7	20.7	17.4, 24.3	
>High school	42.3	38.5, 46.2	23.5	20.4, 26.9	34.3	30.8, 37.9	
**Household Income**							<.0001
<20 k	53.4	46.6, 60.1	27.2	21.7, 33.6	19.4	14.5–25.5	
20 k-<40 k	53.6	47.6, 59.6	26.1	21.1, 31.8	20.3	15.8–25.6	
40 k-<60 k	47.3	40.3, 54.4	24.4	18.8, 31.1	28.3	22.6–34.9	
≥60 k	40.5	36.4, 44.7	23.6	20.2, 27.4	35.9	32.0–40.1	
**Geography**							0.06
Non-Metropolitan Area	40.4	33.5, 47.6	31.2	24.9, 38.2	28.4	22.4, 35.4	
Metropolitan Area	48.0	44.9, 51.1	23.7	21.2, 26.4	28.3	26.7, 31.2	
**Children in Home**							0.23
No children	48.1	44.6, 51.6	25.0	22.1, 28.2	26.9	24.0, 30.0	
Children	44.0	39.3, 48.8	24.6	20.7, 29.1	31.4	27.0, 36.1	
**Regular Health Care provider**							*p* = 0.003
No regular provider	54.5	47.8, 61.0	26.1	20.7, 32.4	19.4	14.7, 25.3	
Regular provider	44.7	41.6, 47.8	24.7	22.1, 27.6	30.6	27.8, 33.6	

TESS Survey 2011.

a MRSA Awareness is having heard of MRSA but unable to define MRSA.

b MRSA Knowledge is the ability to define MRSA.

### Draining Vignette vs. Non-draining vignette

 There was not a significant difference in care seeking among those with lesions that resembled the draining vignette compared to those with lesions that resembled the non-draining vignette. The likelihood of self-treatment for a hypothetical lesion was slightly lower among those who had a draining vignettes compared to those with a non-draining vignette (draining mean 4.4 vs. non-draining mean 4.7; *p* = 0.04) (Table 4). Additionally, those who received the draining vignette were significantly more likely to report that they would cover the lesion (67.4% vs. 57.7%, *p*<0.001) and avoid sharing personal items (67.2% vs. 55.1%, *p*<0.001) than those who received the non-draining vignette.

### Determinants of Self-Care Behaviors and Intention to Self-Treat

Reported intention to use at least one the three specific self-care behaviors that may affect MRSA transmission varied by socio-demographic characteristics. However, the only significant demographic predictor of avoiding sharing personal items was having children in the home (*p* = 0.02) (Table 5). MRSA knowledge was significantly related to all three self-care behaviors; those who reported knowledge of MRSA were more likely to report intentions to use behaviors associated with lower transmission risk: covering a lesion (*p*<0.01) and avoiding sharing personal items (*p*<0.001).

**Table 5:** Weighted Percentages of Affirmative Reponses to Intention to Self-Treat a MRSA-like Sore and Use of Specific Self Care Behaviors.

	Likely to Self-Treat (Weighted Means of 9-point scale)	Cover Sore (Weighted %)	Avoid sharing personal items (Weighted %)	Squeeze pus from Sore (Weighted %)
**All**	4.59	62.53	61.11	55.01
***SOCIO-DEMOGRAPHIC FACTORS***				
**Gender**				
Female	4.46	63.86	63.16	48.01
Male	4.73	61.12	58.93	62.50
	F = 3.66, t = −1.91, *p* = 0.05	*p* = 0.32	*p* = 0.14	*p*<0.001
**Race**				
Non-Hispanic White	4.54	67.17	61.00	54.09
Non-Hispanic Black	4.39	54.91	61.83	53.28
Hispanic	4.76	41.68	57.34	61.05
Non-Hispanic Other	5.03	71.93	68.83	54.79
	F = .93, t = 1.66, *p* = .33	*p*<0.001	*p* = 0.31	*p* = 0.35
**Age**				
18–29 yrs.	4.84	63.65	57.54	62.95
30–44 yrs.	4.69	60.14	62.44	58.79
45–59 yrs.	4.70	63.80	62.17	55.55
≥60 yrs.	4.15	62.67	61.67	43.43
	F = 8.69, t = −2.95, *p* ^trend^ = 0.01	*p* ^trend^ 0 = .91	*p* ^trend^ = 0.40	*p* ^trend^<0.001
**Education**				
≤High School	4.69	58.2	59.8	56.9
>High School	4.52	65.9	62.1	52.2
	F = 5.67, t = −2.38, *p* = 0.07	*p* = 0.005	*p* = 0.41	*p* = 0.13
**Health care provider**				
No regular physician	5.06	65.49	58.59	60.37
Regular Physician	4.47	61.82	61.89	53.96
	F = 11.79, *p*<0.001	*p* = 0.30	*p* = 0.37	*p* = 0.09
**Income**				
<20 k	4.84	49.12	58.98	52.95
20 k-<40 k	4.79	63.20	63.02	53.25
40 k-<60 k	4.88	64.90	58.16	52.13
≥60 k	4.29	66.28	62.04	55.01
	F = 10.43, *p* ^trend^<0.001	*p* ^trend^<0.001	*p* ^trend^<0.68	*p* ^trend^<0.15
**Geography**				
Non Metropolitan Area	4.82	68.14	62.43	51.44
Metropolitan Area	4..54	61.49	60.86	55.70
	F = 2.15, *p*_ = _0.09	*p* = 0.07	*p* = 0.68	*p* = 0.28
**Children in Home**				
No children in Home	4.52	62.74	58.84	54.16
Children in Home	4.73	62.11	65.79	56.78
	F = 2.11, *p* = 0.15	*p* = 0.83	*p* = 0.02	*p* = 0.39
***MRSA-RELATED FACTORS***				
**MRSA knowledge**				
MRSA Unaware	4.58	58.43	57.52	56.35
MRSA Aware	4.75	63.70	58.44	58.23
MRSA Knowledge	4.42	68.62	70.17	50.22
	F = 1.89, *p* = 0.15	*p*<0.01	*p*<0.001	*p* = 0.08
**Received draining vignette**				
Non-Draining	4.73	57.71	55.05	55.96
Draining	4.44	67.35	67.16	54.07
	F = 4.18, *p* = 0.04	*p*<0.001	*p*<0.001	*p* = 0.51
**Lifetime Prevalence of sore**				
No previous lesion	4.40	63.93	61.61	54.06
Previous lesion	5.80	55.31	59.72	62.40
	F = 54.78, *p<0.001*	*p* = 0.03	*p* = 0.63	*p* = 0.04
**Knew someone who had MRSA** a				
No	4.74	64.19	63.61	58.24
Yes	4.39	69.13	65.90	48.01
	F = 3.49, *p* = 0.06	*p* = 0.19	*p* = 0.56	*p* = 0.01
**Likelihood of Sore being MRSA**				
Sore is not MRSA^b^	5.42	59.8	60.3	57.9
Sore is MRSA^c^	4.22	69.2	64.0	50.3
	F = 16.72, *p*<0.001	P0 = .04	p = 0.46	p = 0.13

a Among those who were aware of MRSA and those who could define MRSA.

b Those who responded 1(very unlikely) through 3 to the likelihood of the sore being MRSA.

c Those who responded 7 through 9 (very likely) to the likelihood of the sore being MRSA.

In univariate analyses, the likelihood of self-treatment was higher for respondents who were younger in age (*p* ^trend^ = 0.01), did not have a regular health care provider (*p* = 0.001) or had a history of an SSTI (*p* = 0.001). The intention to self-treat decreased as household income increased (*p* ^trend^<0.001). Among those with awareness or knowledge of MRSA, the likelihood of self-treating was lower among those who believed the lesion was caused by MRSA (*p*<0.001).

A stepwise multinomial logistic model indicates, after adjusting for, gender, age, race, ethnicity, education, income and having a regular health care provider, that those with a previous lesion were twice as likely to self-treat (RR = 2.09 95% CI 1.41, 3.01; *p*<0.004) and half as likely to seek care (RR = 0.53, 95% CI 0.34, 0.82; *p* = 0.004), compared to those who were noncommittal as to whether they would seek care or self-treat (Table 6). Women were less likely to self-treat (RR = 1.37; 95%CI 1.04, 1.80, *p* = 0.02). Increasing age strongly predicted increased care-seeking. Having a lower income was associated with a higher intent to self-treat (RR = 1.72; 95%CI 1.07, 2.76 *p* = 0.03). Those with a regular physician were less likely to self-treat (RR 0.64; 95%CI 0.44, 0.95, *p* = 0.03).

**Table 6:** Multinomial Regression Analysis of Intention to Self-treat.

Variables	Intention to Self-treat				
	More likely to seek care	*p*-values	Equally likely to seek care or self-treat	More likely to self-treat	*p*-values
	RR (95% CI)			RR (95% CI)	
**Lesion History**					
No previous lesion	1.00 (Ref)		1.00	1.00 (Ref)	
Previous lesion	0.53 (0.34, 0.82)	0.004	1.00	2.09 (1.40, 3.07)	<0.001
**Female**	1.37 (1.04, 1.80)	0.02	1.00	1.14 (0.84, 1.53)c	0.42
**Age (per year)**	1.02 (1.01, 1.92)	<0.001	1.00	1.01 (1.00, 1.02)	0.005
**Race/Ethnicity**					
White	1.00 (Ref)		1.00	1.00 (Ref)	
Black	1.35 (0.95, 1.92)	0.10	1.00	1.02 (0.68, 1.55)	0.92
Hispanic	0.99 (0.68, 1.45)	0.98	1.00	0.81 (0.53, 1.22)	0.41
Other	0.83 (0.41, 1.67)	0.60	1.00	1.10 (0.58, 2.07)	0.78
**Education**					
<High School	1.00 (Ref)		1.00	1.00 (Ref)	
High School graduate plus	0.97 (0.72, 1.29)	0.83	1.00	0.80 (0.57, 1.12)	0.19
**Income**					
<20 k	0.94 (0.61, 1.45)	0.81	1.00	1.89 (1.21, 2.95)	0.03
20 k-<40 k	0.77 (0.53, 1.09)	0.15	1.00	1.10 (0.72, 1.67)c	0.67
40 k-<60 k	0.70 (0.47, 1.03)	0.07	1.00	1.10 (0.70, 1.77)c	0.69
≥60 k	1.00 (Ref)		1.00	1.00 (Ref)	
**Health Care Provider**					
No Regular Health Care Provider	1.00 (Ref)		1.00	1.00 (Ref)	
Regular Health Care Provider	1.15 (0.77, 1.70)	0.50	1.00	0.64 (0.44, 0.95)	0.03

## Discussion

This nationally representative survey suggests that estimating the occurrence of SSTIs typical of CA-MRSA just from those presenting with lesions at ambulatory care settings would underestimate the true occurrence of SSTIs in the population and probably misrepresent the descriptive epidemiology of SSTIs. We found that about half of individuals reporting a lifetime history of an SSTI similar to a moderately severe CA-MRSA infection had not sought medical care. Similarly, about half of individuals with such a lesion in the subsequent six-month period also reported that they had not sought medical care. MRSA knowledge was not related to care-seeking. Therefore there is no evidence here to suggest that the reported increase in the numbers of SSTIs, based on clinical encounter data is an artifact of increased awareness of the seriousness of MRSA in the United States [8].

We believe these data represent the first nationwide estimates of the occurrence of SSTIs typical of CA-MRSA lesions in the United States among adults, as well as care-seeking behaviors related to these SSTIs. While our findings cannot be used to precisely adjust the numbers seen in the clinical setting to determine true population incidence, they do suggest that roughly twice the number of infections that present in clinical settings are occurring in the population. To date there are no studies that try to elucidate the proportion of SSTIs that are not brought to the attention of a health care provider.

Our findings of lifetime history and six-month incidence are not consistent with each other: lifetime history reported in the first survey (13%) was double the subsequent six-month occurrence (6%), although one would expect it to be much larger. There are a number of possible explanations. The first may be recall bias: respondents may have underreported SSTIs in the first survey because these may not be memorable health events. The other possibility is that a secular increase in the incidence of CA-MRSA SSTIs in part explains this striking contrast. That is, if a large majority of these infections have occurred relatively recently, then lifetime history might more closely reflect recent history. The first survey may have heightened awareness of SSTIs and contributed to more complete recall six months later (or perhaps even over-identification of lesions during the six-month period). It could also be that the lesions recur reasonably often for the same people and that a relatively large proportion of those who have had such lesions in recent years had a recurrence during the six month period [11]. In other words, because the incidence of such infections is not purely random, one would not interpret the proportion of infections during the six months to suggest that a different 6 percent of the population would get such infections during each six-month period. In any case, the six-month data suggest very high rates of SSTIs similar to CA-MRSA infections in the general U.S. population. Extrapolating from the six-month data and assuming 0–100 percent recurrence in the next six months, somewhere between 6 percent and 12 percent of the U.S. population would have an SSTI similar to those described in our vignettes annually. If the non-responders to the second survey wave were all assumed to have not had an infection, then the estimate would be 4 percent to 8 percent.

The adjusted multinomial regression suggests that intention to self-treat was approximately twice as high among those with experience of a previous lesion. Why previous lesions actually decrease the likelihood of seeking professional care is unclear, although those whose sores resolved without complications may feel there is no gain to professional care. In addition, those without a regular doctor were significantly more likely to self-treat, and it is estimated that about one in five in the United States do not have a usual source of care [12]. Thus reported socioeconomic disparities in MRSA incidence likely underestimate true disparities.

Increased MRSA knowledge did not increase the likelihood of seeking care, although there was a trend towards significance for knowing someone who had had a MRSA infection being more likely to seek care (among respondents who were aware of MRSA). However, MRSA knowledge was strongly associated with the use of self-care behaviors likely to reduce MRSA transmission (covering an SSTI, not squeezing pus from an SSTI and avoiding sharing personal items with household members) suggesting there is a role for public health information to affect transmission of CA-MRSA.

This study has limitations that should be acknowledged. Respondents' reported SSTIs may have been caused by conditions other than MRSA. However our estimate that about 50% of those with similar SSTIs sought care should apply to all etiologies of similarly severe sores. We only assessed prior history of SSTIs typical of CA-MRSA infection by self-report, and we do not know if the respondents' previous SSTIs were due to CA-MRSA. While the description was of a moderately severe SSTI typical of MRSA; milder SSTIs may also be caused by MRSA and would not have been captured in our survey. Furthermore, the self-report of lesions may be affected by recall bias, respondents with comorbidities or other symptoms may be more likely to recall previous lesions which may also be related to care-seeking. Because of the relatively low incidence of infection, we are primarily examining specific self-care behaviors for a hypothetical SSTI and cannot necessarily assume that these behaviors would be the same in the event that a respondent was faced with an actual SSTI. Although the internet panel is based on United States Postal Service's Computerized Delivery Sequence file, there may be differences among those who agree to take part in the panel; however recruitment of participants is done across a large population and may capture a greater proportion of the population compared to telephone sampling. Our survey oversamples African American and Hispanic individuals, allowing estimates of group rates. Furthermore, while SSTIs have various etiologies, the study highlights that approximately half of those with SSTIs do not seek care. We are unaware of any other data about the occurrence of SSTIs among a nationally representative sample in the United States.

## Conclusions

Our data suggest the magnitude of SSTI incidence in the population is greater than estimates based on patients who have sought medical care. We found that roughly 50% of people with a moderately severe SSTI said that they had or would care for such a lesion themselves and not seek medical care. A large proportion of the population is unaware of MRSA and only about a quarter of adults in the U.S. could define MRSA. Intention to self-treat was not influenced by knowledge of MRSA, and thus increasing knowledge of MRSA is unlikely to explain much of the apparent increase in incidence in the United States. While knowing what MRSA is does not seem to be associated with seeking care, it is associated with self-care behaviors that may reduce the transmission of MRSA, and thus public health informational campaigns could reduce transmission.

## Supporting Information

File S1 **Survey.**
(DOC)
