# Supplementary material for: A National Survey of Skin Infections, Care Behaviors and MRSA Knowledge in the United States
Source: PLoS One. 2014 Aug 19;9(8):e104277. doi: 10.1371/journal.pone.0104277 (PMC4138108; doi:10.1371/journal.pone.0104277)
Supplement: File S1 — Survey. (DOC) [file pone.0104277.s001.doc]

TESS2 081 - Wegener

July 2011

- Study Details -

Note: This page may be removed when the questionnaire is sent to the client. However, it must exist in the version sent to Operations.

| **SNO** | **15158** |
| --- | --- |
| **Survey Name** | **TESS2 - 081 Wegener TIME 1** |
| **Client Name** | **University of Chicago** |
| **Great Plains Project Number** | **TBD** |
| **Project Director Name** | **Nukulkij** |
| **Team/Area Name** | **S2** |

| **Sample Criteria** |  |
| --- | --- |
| **Samvar**  (Include name, type and response values. “None” means none. Blank means standard demos. This must match SurveyMan.) | **XTESS081 (1-4)**  **XPARTY7** (1 Strong Republican; 2 Not Strong Republican; 3 Leans Republican; 4 Undecided/Independent/Other; 5 Leans Democrat; 6 Not Strong Democrat; 7 Strong Democrat; 9 Missing)  **XIDEO** (1 Extremely liberal; 2 Liberal; 3 Slightly liberal; 4 Moderate, middle of the road; 5 Slightly conservative; 6 Conservative; 7 Extremely conservative; 9 Missing)  **XREL1** (1 Baptist—any denomination; 2 Protestant (e.g., Methodist, Lutheran, Presbyterian, Episcopal); 3 Catholic; 4 Mormon; 5 Jewish; 6 Muslim; 7 Hindu; 8 Buddhist; 9 Pentecostal; 10 Eastern Orthodox; 11 Other Christian; 12 Other non-Christian, please specify; 13 None; 14 Missing)  **XREL2** (1 More than once a week; 2 Once a week; 3 Once or twice a month; 4 A few times a year; 5 Once a year or less; 6 Never; 9 Missing) |
| **Specified Pre-coding Required** |  |
| **Timing Template Required** (y/n) | **Enabled by default** |
| **Multi-Media** | **YES** |

Note: The change request log can be deleted, if you do not require it.

| **Change Request Log**  (Operations Please Disregard)  Note: Do not change Question numbers after Version 1; to add new question, use alpha characters (e.g., 3a, 3b, 3c) | | | | | |
| --- | --- | --- | --- | --- | --- |
| Author | Ver- sion | Description of Change  (Q#, plus change) | Approval Name | Date Apprv’d | Com-pleted (Y/N) |
|  |  |  |  |  |  |

**Important: Do not change Question numbers after Version 1; to add a new question, use alpha**

**characters (e.g., 3a, 3b, 3c.) Changing question numbers will cause delays and**

**potentially errors in the program.**

TESS2 081 - Wegener

July, 2011

- Questionnaire -

**BEHAVIORAL REACTIONS TO SKIN INFECTIONS: INFORMING AGENT-BASED MODELING OF THE MRSA EPIDEMIC**

**PI Contact Information:**

**Duane Wegener**

**Ohio State University**

**wegener.1@osu.edu**

**614-292-1866**

**Department of Psychology**

**1835 Neil Avenue**

**Columbus, OH 43210**

**Design:**

**The experiment is a 2 (Draining vs. Non-draining sore) X 2 (Message vs. No Message) between-subjects design.**

**Disadvantaged populations are more likely to have MRSA infections than the general public. Therefore, African American and Latino populations will play key roles in the agent-based models these data will inform. We would like to oversample from these groups such that 20% of our sample would be African American, 20% Latino, and 60% from other groups (i.e., White and other). These are not extreme oversamples (approximately twice the incidence in the population), so we are hoping that they would not result in much decrease in the maximum number of participants allotted to the study. We will need a substantial number of respondents from each racial group within each of our sore-type and message conditions in order to provide reliable estimates of parameters for our agent-based population models (especially to enable us to incorporate the other demographic variables within each of the racial groups). Based on the number of items in the proposed study, the maximum N would be approximately 1100 (assuming that half of our participants receive 18 items – in the treatment group – and the other half receive 17 items – in the control group). However, between 25 and 50% of participants will likely say that they have not heard of MRSA in item #8. Those participants will not answer items 9 or 10, so those participants would actually only receive 16 items if in the treatment group or 15 items if in the control group. Then the average number of items across all participants would be just below 17, for a maximum N between 1150 and 1200. We understand that the oversampling of racial groups might reduce N, but we would like to keep the N above 1000 if possible.**

**The only potential special stimulus requests would be the image of a red circle (labeled with the size of the sore in the description) that accompanies the description of the sore. The description is quite short, so a single image could include both the description and circle. It is included below in Word format along with the other items, but we could easily send a PDF or other image format if that would be preferred. We realize that there is likely to be variation across computer screens in the size of the image, but we would like for the pictured circle to be as close to the labeled size (i.e., 1.5 inches) as possible.**

**Sample: n= [200 African American participants + 200 Latino participants + 600 White and other participants] with [18] items each for total of [18,000] respondent-items.**

**ITEMS REQUESTED FROM PROFILE DATA, ADD:**

**We would like to receive information on the participant’s general physical health status (pph10001), whether the participant has one doctor that they usually go to for medical care (pph10003), and whether the person thinks that the greatest impact on overall health comes from genetics, environment, health habits, preventive health services, or something else (pph10231).**

**Description of Study:**

**Draining sore version (SHOW IF XTESS081=1 or 2)**

**[DISPLAY]**

The following research materials address people’s reactions and responses to skin infections. The study involves reading about a particular skin infection and answering questions about how you would react to that kind of infection if you had it on your skin. The questions will take about five minutes to complete. Any responses you provide will be kept confidential. The responses provided to the researchers will not be associated with any information that would identify you as the respondent. You can stop the study at any time without penalty or can skip any questions that you prefer not to answer. You can direct any questions about the research to the Principal Investigator, Dr. Duane T. Wegener at the Ohio State University (email: Wegener.1@osu.edu; phone: 614-292-1866). For questions about your rights as a participant in this study or to discuss other study-related concerns or complaints with someone who is not part of the research team, you may contact Ms. Sandra Meadows in the OSU Office of Responsible Research Practices at 1-800-678-6251. This research is sponsored in part through a grant from the National Science Foundation to fund Time-sharing Experiments in the Social Sciences (TESS).

**[DISPLAY]**

1.5 Inches

Imagine that you have a red, painful, warm sore on your skin that is about 1.5 inches across. The sore hurts more when you touch it and is starting to ooze thick pus (white liquid). You have had the sore for a few days, and it seems to be getting worse not better.

**[SP]**

Q1a. Have you ever had a sore like this? If “yes” what did you do?

No, I have never had a sore like that

Yes, I left it alone or cared for it myself

Yes, I went to the emergency room

Yes, I went to my personal doctor or clinic

**[GRID; SP]**

Q2a. The consequences of a sore like this are probably:

| Not at all serious |  |  |  |  |  |  |  | Very serious |
| --- | --- | --- | --- | --- | --- | --- | --- | --- |
| 1 | 2 | 3 | 4 | 5 | 6 | 7 | 8 | 9 |

**[GRID; SP]**

Q3a. How easy would it be for you to get a doctor or other medical professional to evaluate (and, if necessary, treat) this type of sore within 48 hours?

| Very easy |  |  |  |  |  |  |  | Very difficult |
| --- | --- | --- | --- | --- | --- | --- | --- | --- |
| 1 | 2 | 3 | 4 | 5 | 6 | 7 | 8 | 9 |

**[GRID; SP]**

Q4a. How likely would you be to treat a sore like that yourself? That is, how likely would you be to deal with the sore without going to get professional care?

| Not at all likely |  |  |  |  |  |  |  | Very likely |
| --- | --- | --- | --- | --- | --- | --- | --- | --- |
| 1 | 2 | 3 | 4 | 5 | 6 | 7 | 8 | 9 |

**[MP]**

Q5a. If you care for this type of sore yourself, which of the following would you do?

Cover it

Try to squeeze out the pus

Avoid sharing personal items like towels with household members

**[SP]**

Q6a. Think back to when a doctor has prescribed an antibiotic for an infection, such as a sore throat or urinary tract infection. How do you usually react?

I have never been prescribed an antibiotic

I do not always fill the prescription

I fill it and take the medicine until I feel better

I fill the prescription and finish all of the medicine even if I feel better while there are still some doses left

**[SP; Prompt once]**

Q7a. Have you ever heard of MRSA (pronounced “Mersa”)?

No

Yes, but I do not know what it is

Yes, it is [fill in a few words to describe MRSA]: ___________

**[SP GRID; SHOW Q8a IF Q7a=2 OR 3]**

Q8a.

If you have a painful skin sore of the type described earlier, how likely is it that the sore is MRSA (antibiotic-resistant staph infection)?

| Not at all likely |  |  |  |  |  |  |  | Very likely |
| --- | --- | --- | --- | --- | --- | --- | --- | --- |
| 1 | 2 | 3 | 4 | 5 | 6 | 7 | 8 | 9 |

**[SP; SHOW Q9a IF Q7a=2 OR 3]**

Q9a. How many people (not counting yourself) have you known who had MRSA?

None
1
2
3
More than 3

**[DISPLAY; SHOW IF XTESS081=2]**

The Centers for Disease Control provide the following information about MRSA:

MRSA is methicillin-resistant *Staphylococcus aureus*, a potentially dangerous type of staph bacteria that is resistant to certain antibiotics and may cause skin and other infections. You can get MRSA through direct contact with an infected person or by sharing personal items, such as towels or razors that have touched infected skin.

What are the signs and symptoms of MRSA skin infections? Most staph skin infections, including MRSA, appear as a bump or infected area on the skin that may be:

- Red
- Swollen
- Painful
- Warm to the touch
- Full of pus or other drainage
- Accompanied by a fever

If you or someone in your family experiences these signs and symptoms, cover the area with a bandage and contact your healthcare professional. It is especially important to contact your healthcare professional if signs and symptoms of a MRSA skin infection are accompanied by a fever.

**[GRID; SP]**

Q10a. How likely would you be to seek medical care if you had a red, painful, sore on your skin that is about 1.5 inches across and is starting to ooze thick pus (white liquid)?

| Not at all likely |  |  |  |  |  |  |  | Very likely |
| --- | --- | --- | --- | --- | --- | --- | --- | --- |
| 1 | 2 | 3 | 4 | 5 | 6 | 7 | 8 | 9 |

**[GRID; SP]**

Q11a. If left untreated, the consequences of a red, painful, sore on your skin that is about 1.5 inches across and is starting to ooze thick pus are probably:

| Not at all serious |  |  |  |  |  |  |  | Very serious |
| --- | --- | --- | --- | --- | --- | --- | --- | --- |
| 1 | 2 | 3 | 4 | 5 | 6 | 7 | 8 | 9 |

**[GRID; SP]**

Q12a. If you had a red, painful, sore on your skin that is about 1.5 inches across and is starting to ooze thick pus, how likely is it that the sore would be MRSA (antibiotic-resistant staph infection)?

| Not at all likely |  |  |  |  |  |  |  | Very likely |
| --- | --- | --- | --- | --- | --- | --- | --- | --- |
| 1 | 2 | 3 | 4 | 5 | 6 | 7 | 8 | 9 |

**Non-draining sore version (SHOW IF XTESS081=3 or 4)**

**[DISPLAY]**

Hello. The following research materials address people’s reactions and responses to skin infections. The study involves reading about a particular skin infection and answering questions about how you would react to that kind of infection if you had it on your skin. The questions will take about five minutes to complete. Any responses you provide will be kept confidential. The responses provided to the researchers will not be associated with any information that would identify you as the respondent. You can stop the study at any time without penalty or can skip any questions that you prefer not to answer. You can direct any questions about the research to the Principal Investigator, Dr. Duane T. Wegener at the Ohio State University (email: Wegener.1@osu.edu; phone: 614-292-1866). For questions about your rights as a participant in this study or to discuss other study-related concerns or complaints with someone who is not part of the research team, you may contact Ms. Sandra Meadows in the OSU Office of Responsible Research Practices at 1-800-678-6251. This research is sponsored in part through a grant from the National Science Foundation to fund Time-sharing Experiments in the Social Sciences (TESS).

**[DISPLAY]**

1.5 Inches

Imagine that you have a red, painful, warm sore on your skin that is about 1.5 inches across. The sore is firm to the touch, hurts more when you touch it, and feels like there might be pus under the skin. You have had the sore for a few days, and it seems to be getting worse not better.

**[SP]**

Q1b. Have you ever had a sore like this? If “yes” what did you do?

No, I have never had a sore like that

Yes, I left it alone or cared for it myself

Yes, I went to the emergency room

Yes, I went to my personal doctor or clinic

**[GRID; SP]**

Q2b. The consequences of a sore like this are probably:

| Not at all serious |  |  |  |  |  |  |  | Very serious |
| --- | --- | --- | --- | --- | --- | --- | --- | --- |
| 1 | 2 | 3 | 4 | 5 | 6 | 7 | 8 | 9 |

**[GRID; SP]**

Q3b. How easy would it be for you to get a doctor or other medical professional to evaluate (and, if necessary, treat) this type of sore within 48 hours?

| Very easy |  |  |  |  |  |  |  | Very difficult |
| --- | --- | --- | --- | --- | --- | --- | --- | --- |
| 1 | 2 | 3 | 4 | 5 | 6 | 7 | 8 | 9 |

**[GRID; SP]**

Q4b. How likely would you be to treat a sore like that yourself? That is, how likely would you be to deal with the sore without going to get professional care?

| Not at all likely |  |  |  |  |  |  |  | Very likely |
| --- | --- | --- | --- | --- | --- | --- | --- | --- |
| 1 | 2 | 3 | 4 | 5 | 6 | 7 | 8 | 9 |

**[MP]**

Q5b. If you care for this type of sore yourself, which of the following would you do?

Cover it

Try to squeeze out the pus

Avoid sharing personal items like towels with household members

**[SP]**

Q6b. Think back to when a doctor has prescribed an antibiotic for an infection, such as a sore throat or urinary tract infection. How do you usually react?

I have never been prescribed an antibiotic

I do not always fill the prescription

I fill it and take the medicine until I feel better

I fill the prescription and finish all of the medicine even if I feel better while there are still some doses left

**[SP; prompt once]**

Q7b. Have you ever heard of MRSA (pronounced “Mersa”)?

No

Yes, but I do not know what it is

Yes, it is [fill in a few words to describe MRSA]: ___________

**[SP GRID; SHOW Q8b IF Q7b=2 OR 3]**

Q8b. If you have a painful skin sore of the type described earlier, how likely is it that the sore is MRSA (antibiotic-resistant staph infection)?

| Not at all likely |  |  |  |  |  |  |  | Very likely |
| --- | --- | --- | --- | --- | --- | --- | --- | --- |
| 1 | 2 | 3 | 4 | 5 | 6 | 7 | 8 | 9 |

**[SHOW Q9b IF Q7b=2 OR 3]**

Q9b. How many people (not counting yourself) have you known who had MRSA?

None
1
2
3
More than 3

**[DISPLAY; SHOW IF XTESS081=4]**

The Centers for Disease Control provide the following information about MRSA:

MRSA is methicillin-resistant *Staphylococcus aureus*, a potentially dangerous type of staph bacteria that is resistant to certain antibiotics and may cause skin and other infections. You can get MRSA through direct contact with an infected person or by sharing personal items, such as towels or razors that have touched infected skin.

What are the signs and symptoms of MRSA skin infections? Most staph skin infections, including MRSA, appear as a bump or infected area on the skin that may be:

- Red
- Swollen
- Painful
- Warm to the touch
- Full of pus or other drainage
- Accompanied by a fever

If you or someone in your family experiences these signs and symptoms, cover the area with a bandage and contact your healthcare professional. It is especially important to contact your healthcare professional if signs and symptoms of a MRSA skin infection are accompanied by a fever.

**[GRID; SP]**

Q10b. How likely would you be to seek medical care if you had a red, painful, sore on your skin that is about 1.5 inches across and is firm and hurts more when you touch it?

| Not at all likely |  |  |  |  |  |  |  | Very likely |
| --- | --- | --- | --- | --- | --- | --- | --- | --- |
| 1 | 2 | 3 | 4 | 5 | 6 | 7 | 8 | 9 |

**[GRID; SP]**

Q11b. If left untreated, the consequences of a red, painful, sore on your skin that is about 1.5 inches across and is firm and hurts more when you touch it are probably:

| Not at all serious |  |  |  |  |  |  |  | Very serious |
| --- | --- | --- | --- | --- | --- | --- | --- | --- |
| 1 | 2 | 3 | 4 | 5 | 6 | 7 | 8 | 9 |

**[GRID; SP]**

Q12b. If you had a red, painful, sore on your skin that is about 1.5 inches across and is firm and hurts more when you touch it, how likely is it that the sore would be MRSA (antibiotic-resistant staph infection)?

| Not at all likely |  |  |  |  |  |  |  | Very likely |
| --- | --- | --- | --- | --- | --- | --- | --- | --- |
| 1 | 2 | 3 | 4 | 5 | 6 | 7 | 8 | 9 |

[SP]

**Q41.** Based on what you know or have heard which of the following do you think has the greatest impact on a person’s overall health?

Genetics or heredity 1

The environment 2

Individuals’ health habits (such as diet, exercise, etc.) 3

Use of preventive health services (such as annual physical exams and screening tests) 4

Something else 5

Not sure 6

PARTY7

Show PARTY1 if XPARTY7 = 9 (missing).

[SP]

PARTY1. Generally speaking, do you think of yourself as a...

Republican 1

Democrat 2

Independent 3

Another party, please specify: _____ 4

No preference 5

Ask PARTY2 if “Republican” at PARTY1.

[SP]

PARTY2. Would you call yourself a...

Strong Republican 1

Not very strong Republican 2

Ask PARTY3 if “Democrat” at PARTY1.

[SP]

PARTY3. Would you call yourself a...

Strong Democrat 1

Not very strong Democrat 2

Ask PARTY4 if “Independent”, “Another party”, or “No preference” or skip at PARTY1.

[SP]

PARTY4. Do you think of yourself as closer to the...

Republican Party 1

Democratic Party 2

Data-only

[SP]

DOV_XPARTY7. Merge coding of XPARTY7 and missing data ask.

Strong Republican 1

Not Strong Republican 2

Leans Republican 3

Undecided/Independent/Other 4

Leans Democrat 5

Not Strong Democrat 6

Strong Democrat 7

Refused -1

If XPARTY7≠9 then DOV_XPARTY7=XPARTY7;

Else DOV_XPARTY7=Recoded value as defined by the following:

IF (PARTY1=1 & PARTY2=1) DOV_XPARTY7=1

IF (PARTY1=1 & PARTY2=2) DOV_XPARTY7=2

IF (PARTY1=1 & PARTY2=REFUSED) DOV_XPARTY7=2

IF (PARTY1=3 & PARTY4=1) DOV_XPARTY7=3

IF (PARTY1=4 & PARTY4=1) DOV_XPARTY7=3

IF (PARTY1=5 & PARTY4=1) DOV_XPARTY7=3

IF (PARTY1=REFUSED & PARTY4=1) DOV_XPARTY7=3

IF (PARTY1=3 & PARTY4=2) DOV_XPARTY7=5

IF (PARTY1=4 & PARTY4=2) DOV_XPARTY7=5

IF (PARTY1=5 & PARTY4=2) DOV_XPARTY7=5

IF (PARTY1=REFUSED & PARTY4=2) DOV_XPARTY7=5

IF (PARTY1=2 & PARTY3=1) DOV_XPARTY7=7

IF (PARTY1=2 & PARTY3=2) DOV_XPARTY7=6

IF (PARTY1=2 & PARTY3=REFUSED) DOV_XPARTY7=6

IF (PARTY1=1 & PARTY2=REFUSED) DOV_XPARTY7=2

IF (PARTY1=2 & PARTY3=REFUSED) DOV_XPARTY7=6

IF (PARTY1=3 & PARTY4=REFUSED) DOV_XPARTY7=4

IF (PARTY1=4 & PARTY4=REFUSED) DOV_XPARTY7=4

IF (PARTY1=5 & PARTY4=REFUSED) DOV_XPARTY7=4

IF (PARTY1=REFUSED & PARTY4=REFUSED) DOV_XPARTY7=4

Previously programmed in SNO 12281 (Q7-Q10).

IDEOLOGY

Show IDEO if XIDEO = 9 (missing).

**[SP]**

IDEO. In general, do you think of yourself as…

Extremely liberal 1

Liberal 2

Slightly liberal 3

Moderate, middle of the road 4

Slightly conservative 5

Conservative 6

Extremely conservative 7

[SP]

DOV_IDEO. Merge coding of XIDEO and missing data ask.

Extremely liberal 1

Liberal 2

Slightly liberal 3

Moderate, middle of the road 4

Slightly conservative 5

Conservative 6

Extremely conservative 7

Refused -1

If XIDEO≠9 then DOV_IDEO=XIDEO;

Else DOV_IDEO=IDEO.

RELIGION1

Show REL1 if XREL1= 14 (missing).

[SP]

REL1. What is your religion?

**[Do not rotate]**

Baptist—any denomination 1

Protestant (e.g., Methodist, Lutheran, Presbyterian, Episcopal) 2

Catholic 3

Mormon 4

Jewish 5

Muslim 6

Hindu 7

Buddhist 8

Pentecostal 9

Eastern Orthodox 10

Other Christian 11

Other non-Christian 12

None 13

Prompt once.

[SP]

DOV_REL1. Merge coding of REL1 and missing data ask.

Baptist—any denomination 1

Protestant (e.g., Methodist, Lutheran, Presbyterian, Episcopal) 2

Catholic 3

Mormon 4

Jewish 5

Muslim 6

Hindu 7

Buddhist 8

Pentecostal 9

Eastern Orthodox 10

Other Christian 11

Other non-Christian 12

None 13

Refused -1

If XREL1≠14 then DOV_REL1=XREL1;

Else DOV_REL1=REL1.

RELIGION2

Ask REL2 if REL1 is not “None”.

[SP]

REL2. How often do you attend religious services?

More than once a week 1

Once a week 2

Once or twice a month 3

A few times a year 4

Once a year or less 5

Never 6

[SP]

DOV_REL2. Merge coding of REL2 and missing data ask.

More than once a week 1

Once a week 2

Once or twice a month 3

A few times a year 4

Once a year or less 5

Never 6

If XREL2≠9 then DOV_REL2=XREL2;

Else DOV_REL2=REL2.

Insert standard close.
